# BRCA1 Is Required for Maintenance of Phospho-Chk1 and G_2_/M Arrest during DNA Cross-Link Repair in DT40 Cells

**DOI:** 10.1128/MCB.01497-14

**Published:** 2015-10-16

**Authors:** Margarethe Draga, Elizabeth B. Madgett, Cassandra J. Vandenberg, David du Plessis, Aisling Kaufmann, Petra Werler, Prasun Chakraborty, Noel F. Lowndes, Kevin Hiom

**Affiliations:** a Division of Cancer Research, Medical Research Institute, Jacqui Wood Cancer Centre, Ninewells Hospital and Medical School, Dundee, Scotland; b MRC Laboratory of Molecular Biology, Cambridge Biomedical Campus, Cambridge, United Kingdom; c Genome Stability Laboratory, Centre for Chromosome Biology, School of Natural Science, National University of Ireland, Galway, Ireland; d Institute of Anatomy II, Department of Vertebrate Embryology, University of Cologne, Cologne, Germany

## Abstract

The Fanconi anemia DNA repair pathway is pivotal for the efficient repair of DNA interstrand cross-links. Here, we show that FA-defective (*Fancc*^−^) DT40 cells arrest in G_2_ phase following cross-link damage and trigger apoptosis. Strikingly, cell death was reduced in *Fancc*^−^ cells by additional deletion of the BRCA1 tumor suppressor, resulting in elevated clonogenic survival. Increased resistance to cross-link damage was not due to loss of toxic BRCA1-mediated homologous recombination but rather through the loss of a G_2_ checkpoint. This proapoptotic role also required the BRCA1-A complex member ABRAXAS (FAM175A). Finally, we show that BRCA1 promotes G_2_ arrest and cell death by prolonging phosphorylation of Chk1 on serine 345 after DNA damage to sustain arrest. Our data imply that DNA-induced cross-link death in cells defective in the FA pathway is dependent on the ability of BRCA1 to prolong cell cycle arrest in G_2_ phase.

## INTRODUCTION

The Fanconi anemia (FA) repair pathway is dedicated to the repair of replication blocking lesions, such as interstrand DNA cross-links (ICL) and DNA-protein cross-links, which pose a major threat to the maintenance of genomic stability. Defects in any of 17 FA genes in patients confer the hematological disease Fanconi anemia. Characteristically, defects in any of these genes render patients' cells exquisitely sensitive to DNA cross-linking agents such as mitomycin C, *cis*-diamminedichloridoplatinum (II) (cisplatin), diepoxybutane (1), and reactive aldehydes that cause DNA-protein cross-links (2, 3). The FA pathway is also conserved in organisms as diverse as dictyostelium, nematodes, amphibians, birds, and mammals.

Genetic and biochemical evidence suggests that DNA cross-links represent a strong physical block to replicative polymerases; however, the molecular pathway whereby DNA cross-links are removed and the chromosomes restored by the FA pathway is only partially understood. Eight FA proteins (FANCA, -B, -C, -E, -F, -G, -L, and -M) form a core complex that is recruited to sites of ICL damage, where it ubiquitylates FANCD2 and FANCI (4–6). Although not fully understood, the recruitment of FA proteins to ICLs or to DNA-protein cross-links (DPC) signals for the recruitment of nucleases that incise and unhook DNA cross-links, enabling replication across these noncoding lesions using a translesion polymerase (7, 8). The process is completed with the repair of any resultant DNA breaks through homologous recombination (HR).

Failure to repair DNA cross-link damage in cells defective in the FA pathway results in greatly elevated cell death. Although cell death may arise through failure to complete cell division leading to mitotic catastrophe, a significant proportion occurs through apoptosis following prolonged cell cycle arrest in G_2_ phase. The genetic determinants regulating this proapoptotic response to ICL and DPC damage are poorly understood.

The BRCA1 tumor suppressor protein is known to play a role in DNA cross-link repair in a variety of cell types (4), primarily through its role in promoting HR. However, Bunting et al. recently proposed that BRCA1 has another role in the FA pathway, upstream of its function in HR (9), to facilitate retention of FANCD2 at sites of cross-link repair by influencing local chromatin structure (4, 9, 10).

In addition to its role in promoting repair of DNA damage by HR, BRCA1 has been shown to play a role in ionizing radiation (IR)-induced cell cycle checkpoints at S and G_2_ phases (11). Yarden et al. reported that expression of BRCA1 also restored an IR-induced G_2_ arrest to the BRCA1-defective cancer cell line HCC1937 (12). Moreover, the restoration of G_2_/M checkpoint function correlated with increased activation of Chk1 for the phosphorylation of the cell cycle regulator Cdc25C. In contrast, the p53-dependent G_1_-S cell cycle checkpoint is intact in BRCA1-defective cells (13).

We wanted to understand how BRCA1 might also contribute to cell cycle checkpoint arrest and viability in the FA pathway. To do this, we used the genetically tractable avian cell line DT40, which has previously been established as a good model for FA mediated cross-link repair (10, 14). We report that the lethal effect of cisplatin treatment in *Fancc*^−^ mutant DT40 cells is ameliorated by defects in BRCA1, BARD1, and ABRAXAS (ABRA1). Moreover, we found that increased survival is not linked to repair of lesions by homologous recombination but arises through a failure to maintain cell cycle arrest in G_2_ phase. Together, our data support an important role for BRCA1 to regulate the proliferation of vertebrate cells containing DNA damage and promote genome stability.

## MATERIALS AND METHODS

### Cell culture.

DT40 chicken cells were propagated in standard RPMI medium-supplemented medium at 37°C and 5% CO_2_. Where indicated, 500 μM indole-3-acetic acid was used to deplete BRCA1-DEG protein. The generation of *Abra1*^−/−^ and *Fancc*^−^ *Abra1*^−/−^ DT40 mutant cells was described by D. du Plessis in 2012 (unpublished data).

### Antibodies.

Antibodies against Chk1 (Sc-8408), phosphoserine 345Chk1 (catalog no. 2348), and BRCA1 (Ab-1) were purchased from Santa Cruz Biotech, Cell Signaling, and Calbiochem, respectively.

### Clonogenic survival assay.

Cells were incubated for 1 h at 37°C with medium containing 1 to 40 μM cisplatin as indicated. Cells were washed and suspended in fresh medium, and serial dilutions were plated in medium containing 1% methylcellulose. Where indicated, 2 mM caffeine (Sigma) was included for 1 h prior to addition of cisplatin. Colonies were counted after 8 to 10 days of incubation.

### Cell cycle analysis.

Cells were treated with cisplatin as described above. At different times, the cells were pulsed with 20 μM bromodeoxyuridine (BrdU; Sigma) for 20 min, washed twice with phosphate-buffered saline (PBS), and fixed overnight in 70% ethanol at −20°C. We stained cells with antibody to BrdU conjugated to fluorescein isothiocyanate as previously described (14) and analyzed by using a FACScan cytometer (Becton Dickinson).

### Mitotic index.

A 1 μM concentration of nocodazole (Sigma) was added to cells 15 to 16 h after treatment with cisplatin, followed by incubation for 6 to 7 h. The cells were washed in PBS and fixed overnight in 70% ethanol at −20°C. The cells were stained with rabbit polyclonal antibody to histone H3 (phospho-S10; AbCam) and then with antibody to rabbit conjugated to fluorescein isothiocyanate (Southern Biotechnology) for 30 min, resuspended in PBS containing 25 μg of propidium iodide/ml, and analyzed by using a FACScan cytometer (Becton Dickinson).

### Comet assay.

A modification of alkaline comet assay was performed. Cells were harvested by centrifugation (800 rpm) and kept on ice. Approximately 9 × 10^4^ cells were resuspended in 30 μl of PBS and 300 μl of low-melting-point agarose (LMA; 0.5%) and then placed on a slide percolated with agarose. Cells were lysed using lysis buffer (2.5 mM NaCl, 100 mM EDTA, 10% Tris [pH 10]; 1% Triton and 10% dimethyl sulfoxide were added fresh) for 1 to 2 h at 4°C. Slides were preincubated in alkaline solution for 30 min to 1 h and electrophoresed for 30 min at 21 V (0.86 V/cm) and 300 mA. Slides were neutralized, dried, stained with SYBR green, and analyzed using a fluorescence microscope (LSM 500; Zeiss). Tail moment was measured using Comet Score software. 100 cells per slide were evaluated, and the mean of tail moment (TM) was determined.

### Elutriation.

A total of 3 ×10^8^ DT40 cells were collected by centrifugation and resuspended in 50 ml of PBS plus 1% fetal bovine serum and then drawn into a standard (4-ml) chamber at 2,500 rpm in a Beckman JE-5.0 centrifugal elutriation rotor at a flow rate of 15 ml/min. The cells in G_1_ were harvested by altering the flow rate to 21 ml/min. A 150-ml fraction was collected, and the cells were harvested by centrifugation and then resuspended in fresh RPMI medium.

### MTS assay.

DT40 cells were treated with different concentrations of cisplatin. In one experiment, the cisplatin was washed off after 1 h. Cell lines were plated into 96-well plates at a density of 15,000 cells per well. The plates were put in an incubator for 48 h, and then 20 μl of CellTiter 96 Aqueous One solution reagent (Promega) was added to each well, followed by incubation for 4 h at 37°C and 5% CO_2_. The absorbance was measured at 490 nm.

### Chromosome break analysis.

Chromosome breaks were analyzed by metaphase spread from cells incubated with or without treatment with 1 μM cisplatin for 1 h. After 15 h, colcemid was added for 1.5 h, and the cells were harvested and then incubated with 75 mM KCl for 25 min. The cells were fixed with methanol-acetic acid (3:1) four times and then stored at −20°C. Cells were dropped onto slides and stained with DAPI (4′,6′-diamidino-2-phenylindole). A total of 50 metaphases were scored for each treatment. Scoring was restricted to chicken macrochromosomes only.

## RESULTS

### The lethal effect of ICL damage in *Fancc*^−^ cells is ameliorated by defects in BRCA1/BARD1.

**FIG 1:**
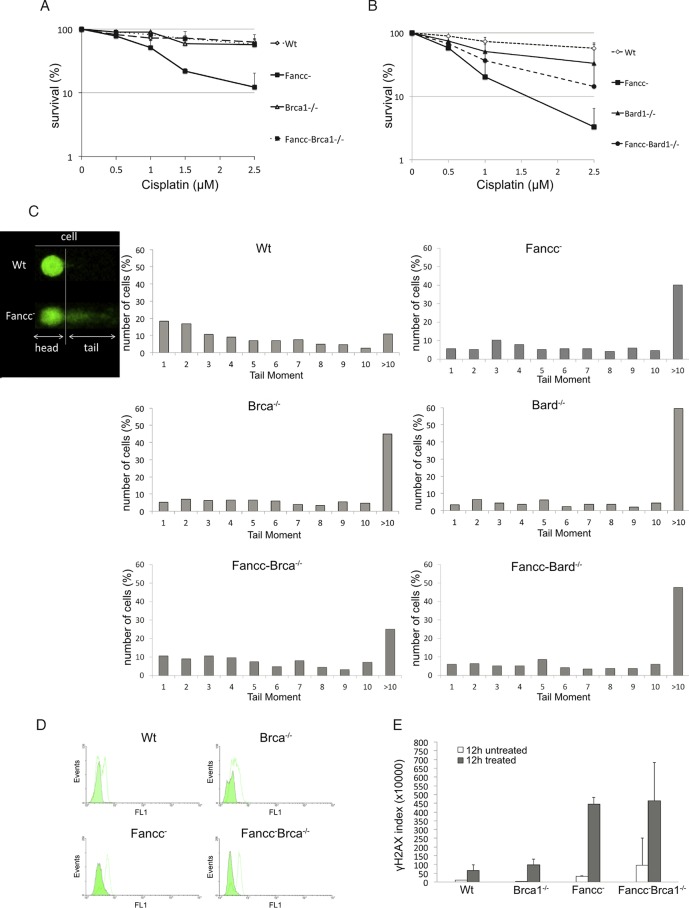
Defects in BRCA1 ameliorate the sensitivity of *Fancc*^−^ DT40 cells to cisplatin treatment. (A and B) Graphs showing clonogenic survival curves for indicated DT40 mutant cells after treatment with different concentrations of cisplatin. *Fancc*^−^ *Brca1*^−/−^ and *Fancc*^−^ *Bard1*^−/−^ are less sensitive to treatment with low doses of cisplatin compared to *Fancc*^−^ cells. (A) Fifty percent inhibitory concentration values (IC_50_): wild-type cells, 3.5 μM; *Fancc*^−^ cells, 0.8 μM; *Brca1*^−/−^ cells, 3.4 μM; and *Fancc*^−^ *Brca1*^−/−^ cells, 3.8 μM. (B) IC_50_ values: wild-type cells, 3.2 μM; *Fancc*^−^ cells, 0.47 μM; *Bard1*^−/−^ cells, 1.1 μM; and *Fancc*^−^ *Bard1*^−/−^ cells, 0.7 μM. The data presented are means from three experiments; error bars indicate one standard deviation. (C) A comet assay showed the increased tail moment and therefore the persistence of DNA breaks in wild-type and mutant DT40 cells, as indicated. Cells were treated with 1 μM cisplatin for 1 h, allowed to recover in fresh medium for 15 h, and then analyzed by alkaline comet assay. Tail moments (TM) were calculated for >100 comets from each sample, and the percentage of cells showing a specific TM was plotted. (D) Mutant cells were treated with 1 μM cisplatin for 1 h, allowed to recover in fresh medium for 12 h, and then stained with primary antibody against γH2AX and secondary antibody conjugated to fluorescein isothiocyanate. γH2AX was quantified by fluorescence-activated cell sorting (FACS), where the FL1 channel corresponds to fluorescein. A green histogram represents untreated cells, and a white histogram represents cells treated with 1 μM cisplatin. (E) Quantification of γH2AX staining in panel D measured in arbitrary units. Further information is contained in Fig. S1 and S3 in the supplemental material.

To investigate the function of BRCA1 in DNA cross-link repair, we compared the cisplatin sensitivity of *Fancc*^−^, *Brca1*^−/−^, and *Fancc*^−^ *Brca1*^−/−^ double-mutant DT40 cells in clonogenic and MTS survival assays. We previously reported that, like cells from FA patients, *Fancc*^−^ and *Brip1*^−/−^ (also called *Fancj*^−/−^) mutant DT40 cells exhibit acute sensitivity to cisplatin-induced DNA damage (14). We confirmed this again for *Fancc*^−^ mutant cells (Fig. 1A). In marked contrast, *Brca1*^−/−^ mutant DT40 were minimally sensitive to treatment with doses of cisplatin up to 2.5 μM in a clonogenic survival assay and more resistant than wild-type cells to cisplatin in a MTS assay (Fig. 1A; see Fig. S1 in the supplemental material) (14). This result was surprising, since other HR-defective DT40 mutants, such as *Xrcc2*^−/−^ cells, are sensitive to ICL damage (10, 15). Nevertheless, we confirmed our findings using multiple clones from two independently constructed BRCA1^−/−^ mutant cell lines (data not shown).

The lack of cisplatin sensitivity observed in BRCA1^−/−^ mutant DT40 contrasts with the cisplatin sensitivity reported for a range of BRCA1-defective mammalian cell lines (16–18). We can think of several reasons why this might be the case. First, BRCA1 might function differently in the repair of cisplatin-induced damage in avian and mammalian cells. Second, differences might reflect the fact that DT40 is a transformed B-cell line compared to most cancer cell lines, which are of epithelial origin. Finally, the sensitivity of cells to DNA damage is profoundly affected by genetic background in different cell lines. For example, whereas some BRCA2 defective human tumors exhibit sensitivity to treatment with DNA cross-linking agents, cells from mice specifically mutated in BRCA2 are largely insensitive to this damage (19). Likewise, the profound sensitivity of BRCA1-defective cells to PARP inhibitors is diminished or lost in cells that also carry a defect in 53BP1.

We previously reported that our *Brca1*^−/−^ mutant cells are impaired in homologous recombination (10, 14, 20). To confirm that this cell line exhibits another important phenotype characteristic of mammalian cells defective in BRCA1, we tested its sensitivity to the PARP inhibitor olaparib. Consistent with the behavior of cells from BRCA1-defective patients and cells from *Brca1*^Δ11/Δ11^ mice, our *Brca1*^−/−^ DT40 mutant was profoundly sensitive to treatment with olaparib. Moreover, sensitivity to olaparib was corrected by expression of a BRCA1 transgene (see Fig. S1 in the supplemental material). Furthermore, as in mammals, sensitivity to olaparib was also partially reversed in *Brca1*^−/−^ mutant DT40 with an additional defect in 53BP1 (21, 22). From this we infer that *Brca1*^−/−^ DT40 cells share fundamental characteristics with mammalian cell lines defective in BRCA1.

Perhaps more surprising than the insensitivity of *Brca1*^−/−^ DT40 to cisplatin treatment was the decreased cisplatin sensitivity observed in *Fancc*^−^ *Brca1*^−/−^ double-mutant cells. These mutant cells exhibited normal survival after treatment with 2.5 μM cisplatin, a dose at which only 5 to 10% *Fancc*^−^ cells survive (Fig. 1A and B). This suggested that loss of BRCA1 function abrogated the toxic effect of 2.5 μM cisplatin in *Fancc*^−^ cells. It is noteworthy that the ameliorating affect of BRCA1 defects in *Fancc*^−^ cells was diminished after treatment with higher concentrations of cisplatin (up to 40 μM), where the burden of DNA lesions is likely sufficient to cause cell death through mitotic catastrophe (see Fig. S1 in the supplemental material).

*In vivo*, BRCA1 functions as a heterodimeric complex with its interacting partner BARD1 (23). Therefore, we next measured clonogenic survival of *Bard1*^−/−^ and *Fancc*^−^ *Bard1*^−/−^ mutant cells treated with cisplatin (Fig. 1B). Like *Brca1*^−/−^ mutant cells, *Bard1*^−/−^ mutant cells were much less sensitive to cisplatin treatment than were *Fancc*^−^ cells. Moreover, the survival was markedly improved in *Fancc*^−^ *Bard1*^−/−^ cells compared to the *Fancc*^−^ single mutant. Hence, the loss of functional BARD1, like the loss of BRCA1, ameliorated the toxic effects of cisplatin in FANCC-deficient cells. However, we note that *Fancc*^−^ *Bard1*^−/−^ mutant cells remained slightly more sensitive to cisplatin than the *Fancc*^−^ *Brca1*^−/−^ compared to a wild-type control.

It was previously reported that inhibition of nonhomologous end joining (NHEJ) suppressed ICL sensitivity in Caenorhabditis elegans mutants defective in the FA pathway, and also in FA-defective DT40 and mammalian cell lines (24, 25). Inhibition of NHEJ in these cells also reduced DNA cross-link-induced chromosome breakage and promoted HR. It was inferred that double-strand breaks (DSBs) generated in the absence of a functional FA pathway are diverted into abortive and toxic repair by NHEJ. Inhibition of NHEJ restored repair fidelity to these cells by permitting more accurate processing of DSB intermediates through HR, resulting in increased cell survival.

We next investigated whether defects in BRCA1 could similarly restore efficient and accurate repair of ICL damage in *Fancc*^−^ mutant cells. To do this, we measured the level of unrepaired breaks in cisplatin-treated cells, using a comet assay (Fig. 1C). We found that treatment with 1 μM cisplatin for 1 h produced an increased comet tail moment in *Fancc*^−^, *Brca1*^−/−^, *Bard1*^−/−^, *Fancc*^−^ *Brca1*^−/−^, and *Fancc*^−^ *Bard1* mutant cells 15 h later compared to a wild-type control. This indicated the persistence of unrepaired DNA damage (Fig. 1C). The distribution of cells with increased tail moment was slightly different in *Fancc*^−^ *Brca1*^−/−^ mutant cells compared to *Fancc*^−^ mutant cells, with the latter comprising more cells with a tail moment of >10. Mutant cells also exhibited increased γH2AX staining compared to wild-type cells (Fig. 1D and E), indicating the persistence of unrepaired DNA lesions. Hence, the improved survival afforded to *Fancc*^−^ cells by defects in BRCA1 or BARD1 could not be explained by enhanced repair of ICL-induced DNA breaks.

### Defects in BRCA1/BARD1 abrogate ICL-induced cell cycle arrest in *Fancc*^−^ cells.

**FIG 2:**
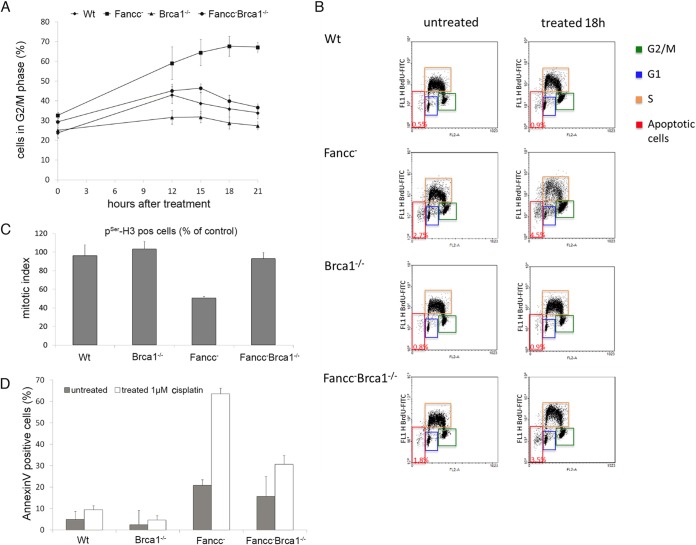
*Fancc*^−^ mutant cells, but not *Brca1*^−/−^ or *Fancc*^−^ *Brca1*^−/−^ cells, arrest with 4C DNA content and undergo apoptotic cell death after treatment with cisplatin. (A and B) Wild-type and mutant cells were damaged by 1 μM cisplatin, pulse-labeled with BrdU, and harvested at the times indicated. Cells were analyzed for BrdU incorporation and propidium iodide staining by FACS to determine the DNA content. The percentages of cells with near 4C DNA content and therefore in G_2_/M phase (green boxes) of the cell cycle are shown at different times after DNA damage treatment. (C) *Fancc*^−^ mutant cells, but not *Brca1*^−/−^ or *Fancc*^−^ *Brca1*^−/−^ cells, arrest prior to entering mitosis after treatment with cisplatin. The mitotic index of cells treated with 1 μM cisplatin is shown. Nocodazole was added 15 h after damage treatment to trap cells entering mitosis. Mitotic cells were quantified 24 h after treatment by staining for pSer-H3 and measured by FACS. The mitotic index is calculated as the ratio of treated to untreated cells staining positive for pSer-H3. (D) Loss of BRCA1 function reduces apoptotic cell death in *Fancc*^−^ mutant cells. The percentage of cells that stained for annexin V, 22 h after treatment 1 μM cisplatin, was used as a measurement of apoptosis. The data shown in panels A, C, and E show the means from three experiments; error bars indicate the standard deviations. See also Fig. S1 in the supplemental material.

Increased resistance to cisplatin induced-damage in *Fancc*^−^ *Brca1*^−/−^ and *Fancc*^−^ *Bard1*^−/−^ cells was not mediated through enhanced repair efficiency. Therefore, we hypothesized that these mutant cells might have an increased tolerance to unrepaired DNA damage. Agents such as cisplatin and mitomycin C are known to cause FA-defective cells to undergo prolonged arrest in G_2_ phase, with a concomitant increase in apoptotic cell death (26, 27). We next investigated the progression of different mutant cells through the cell cycle after cisplatin treatment. Cells were treated with cisplatin for 1 h, and the proportion of cells accumulating in G_2_ phase was measured over time by staining with propidium iodide (Fig. 2A and B). We also measured the accumulation of cells in mitosis (mitotic index) by staining for phospho-histone H3 after addition of nocodazole (Fig. 2C). Lastly, we measured apoptotic cell death by staining for annexin V (Fig. 2D) and by quantifying sub-G_1_ cells stained with propidium iodide (Fig. 2B).

As expected, *Fancc*^−^ mutant DT40 exhibited a profound cell cycle delay in response to cisplatin treatment compared to wild-type cells, with ca. 70% of cells accumulating in G_2_ phase (4C) after 21 h (Fig. 2A). This contrasted markedly with wild-type cells that exhibited a slight increase in G_2_ population approximately 12 to 15 h after cisplatin treatment but returned to starting levels at 21 h (Fig. 2A and B), with little or no increase in apoptosis (Fig. 2D).

These experiments were performed on an asynchronous population of cycling cells. However, different mutant cell lines may exhibit minor differences in cell cycle progression (see Fig. S2 in the supplemental material). To confirm that differences in transit into mitosis after cisplatin treatment was caused by cell cycle arrest/delay and not by inherent differences in cell cycle progression between wild-type and mutant cells, we measured the accumulation of mitotic cells in the presence of the mitotic spindle poison, nocodazole. This permitted us to quantify the accumulation of cells in mitosis several hours after treatment with the DNA-damaging agent and calculate the proportion of the cell population in mitosis after a specific time. Treatment of *Fancc*^−^ cells with cisplatin caused a clear decrease in mitotic index compared to wild-type control, indicating impaired transit of cells into M phase. This is consistent with the execution of a DNA-damage-induced checkpoint at G_2_ phase (Fig. 2C). Furthermore, arrest was accompanied by a marked increase in apoptotic cell death, presumably through the failure of arrested cells to repair DNA damage (Fig. 2B to D).

Despite significant levels of unrepaired DNA breaks, cisplatin-treated *Brca1*^−/−^ and *Bard1*^−/−^ cells progressed through the cell division cycle and into mitosis with very little delay in G_2_. We measured only a small increase in apoptotic cell death for *Brca1*^−/−^ cells (Fig. 2B and D; see Fig. S1 in the supplemental material). The lack of G_2_ arrest was even more apparent when comparing *Fancc*^−^ cells with *Fancc*^−^ *Brca1*^−/−^ and *Fancc*^−^ *Bard1*^−/−^ double mutants. Whereas 70 to 80% *Fancc*^−^ cells accumulated in G_2_ phase 21 h after cisplatin treatment, only 40 to 45% of *Fancc*^−^ *Brca1*^−/−^ and *Fancc*^−^ *Bard1*^−/−^ mutants were in G_2_ (Fig. 2A; see Fig. S1 in the supplemental material). Moreover, we measured no ICL-induced decrease in mitotic index in *Fancc*^−^ *Brca1*^−/−^ mutant cells, a finding consistent with their unhindered progression into mitosis (Fig. 2C). The failure of cisplatin-treated *Fancc*^−^ *Brca1*^−/−^ cells to arrest in G_2_ was accompanied by a significant decrease in apoptotic cell death compared to *Fancc*^−^ cells (Fig. 2B and D). The proliferation of cells with persistent unrepaired DNA damage is consistent with the increased levels of chromosome abnormalities measured in DT40 cells with defects in BRCA1 and FANCC (see Fig. S3 in the supplemental material) (10, 15). Since entry into mitosis with unrepaired DNA damage is characteristic of a defective cell cycle checkpoint, we reasoned that BRCA1 and BARD1 might be required to promote an ICL-induced G_2_/M checkpoint in the FA pathway.

### Defects in ABRAXAS but not BRIP1 abrogate accumulation of cells in G_2_.

Although BRCA1 has been implicated in a G_2_/M checkpoint induced by ionizing radiation, its mechanism of action is unclear (11). However, BRCA1 is known to interact in a cell cycle-dependent manner with the ABRAXAS (Fam175A), BRIP1 (FANCJ), and CtIP proteins, forming the BRCA1-A, BRCA1-B, and BRCA1-C complexes, respectively (28–30). Although CtIP plays an important role in the resection of DSB to promote homologous recombination, BRIP1 and ABRAXAS have been implicated in ionizing radiation (IR)-induced checkpoint function (31–35). We hypothesized that ABRAXAS and/or BRIP1 proteins might contribute to cell cycle arrest in cisplatin-treated FA cells.

**FIG 3:**
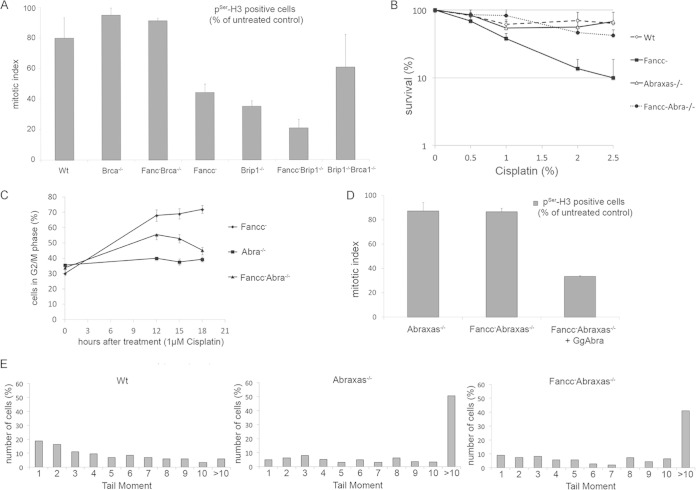
Loss of ABRAXAS but not BRIP1 abrogates G_2_/M arrest in FANCC-defective cells. (A) Mitotic index of mutant cells treated and not treated with 1 μM cisplatin and stained for pSer-H. (B) Clonogenic survival curves for indicated DT40 mutant cells after treatment with different concentrations of cisplatin. *Fancc*^−^ *Abra*^−/−^ mutants are less sensitive to treatment with low doses of cisplatin than *Fancc*^−^ cells. (C) *Fancc*^−^ cells, but not *Abra1*^−/−^ or *Fancc*^−^ *Abra1*^−/−^ cells, arrest in G_2_/M with near 4C DNA content after cisplatin treatment. DNA content was quantified by using FACS after incorporation of BrdU into cells and after staining with propidium iodide. The percentage of cells with 4C content and therefore in G_2_/M phase are shown. (D) Mitotic index of cells treated or not treated with 5 μM cisplatin. The percentage of cells positive for pSer-H3 is shown. The experiments from panels C and D were performed at the same time as those depicted in Fig. 2A and C. The wild-type control in the latter experiments can be used for comparison. (E) A comet assay of mutant cells (as indicated) shows the persistence of DNA strand breaks 15 h after treatment with 1 μM cisplatin. Tail moments were calculated for >100 comets and are shown as a percentage in the total cell population. The data shown in panels A to D are the means from three experiments; error bars indicate the standard deviations.

We previously reported that *Fancc*^−^ *Brip1*^−/−^ double mutant cells are more sensitive to ICL damage than either *Brip1*^−/−^ or *Fancc*^−^ single mutants (14). Accordingly, we found that treatment of *Fancc*^−^ *Brip1*^−/−^ cells with cisplatin caused an even more profound decrease in mitotic index than observed in *Fancc*^−^ mutant cells. We concluded that the G_2_ checkpoint was functioning normally in these cells (Fig. 3A). In contrast, *Fancc*^−^ *Abra1*^−/−^ mutant cells were much less sensitive to cisplatin-induced damage than *Fancc*^−^ cells (Fig. 3B). Moreover, neither *Abra1*^−/−^ nor *Fancc*^−^ *Abra1*^−/−^ mutant cell lines exhibited prolonged accumulation in G_2_ phase after exposure to 1 μM cisplatin (Fig. 3C). Consistent with this, we measured little decrease in mitotic index in *Abra1*^−/−^ or *Fancc*^−^ *Abra1*^−/−^ cells treated with cisplatin (Fig. 3D). This again indicated a failure to arrest in G_2_ despite elevated levels of unrepaired DNA breaks (Fig. 3E). Reintroduction of ABRAXAS into *Fancc*^−^ *Abra1*^−/−^ cells restored cisplatin-induced decrease in mitotic index in these cells, indicating a functional cell cycle arrest. We concluded that, like defects in BRCA1/BARD1, the loss of ABRAXAS function is linked to impaired cell cycle arrest in *Fancc*^−^ mutant cells treated with cisplatin. It is of particular note that, whereas BRCA1 mutant cells are impaired for homologous recombination, human and DT40 cells defective in ABRAXAS are not HR defective (36). This indicates that the ICL-induced checkpoint might be genetically separable from HR.

### BRCA1 and ABRAXAS are required for the maintenance but not the initiation of G_2_ arrest in FA cells.

**FIG 4:**
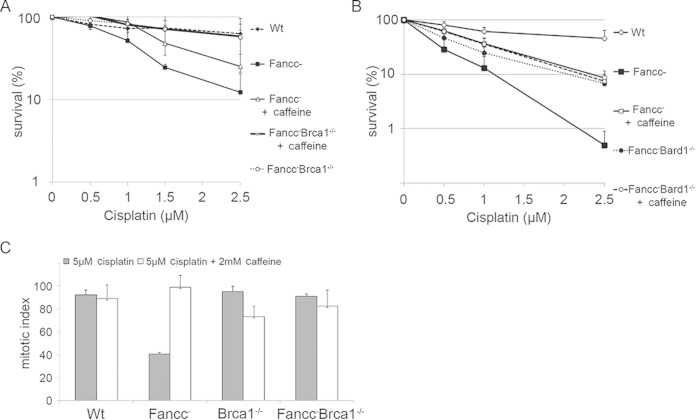
Caffeine treatment improves survival in *Fancc*^−^ DT40 mutant cells treated with cisplatin. (A and B) Clonogenic survival assays were performed for mutant cells treated with different concentrations of cisplatin and in the presence or absence of 2 mM caffeine. The inclusion of 2 mM caffeine in the media improves survival in *Fancc*^−^ cells treated with different concentrations of cisplatin. (C) Mitotic index of cells treated and not treated with 5 μM cisplatin with or without 2 mM caffeine. The percentage of cells positive for pSer-H3 is shown. Caffeine improves cell cycle progression into mitosis in *Fancc*^−^ cells. The data presented are the mean of three experiments; error bars indicate one standard deviation. See also Fig. S4 in the supplemental material.

DNA damage-induced checkpoints are established through the activity of the checkpoint kinases ATM and ATR. Treatment with caffeine, which inhibits the action of these kinases, has previously been shown to modulate G_2_ accumulation in response to X-rays (37). Therefore, we examined the effect of inhibiting these kinases on ICL-induced G_2_ arrest in *Fancc*^−^ cells. We treated wild-type and mutant cells with 1 μM cisplatin for 1 h in the presence or absence of caffeine to inhibit ATM and ATR and measured clonogenic survival and the mitotic index. We found that inclusion of caffeine markedly improved the survival of *Fancc*^−^ cells treated with 0.5 to 2 μM cisplatin (Fig. 4A and B). Survival in caffeine-treated *Fancc*^−^ cells was almost identical to that of *Fancc*^−^ *Bard1*^−/−^ mutant cells. The mitotic index of these cells was also similar to the wild type, an observation consistent with a progression into mitosis without delay (Fig. 4C). Importantly, caffeine had little additional impact on either the survival or mitotic index in *Fancc*^−^ *Bard1*^−/−^ cells. We observed a similar defect in cell cycle arrest upon treatment of *Fancc*^−^ cells with specific inhibitors of ATM and ATR, i.e., KU55933 and ETP 46464, respectively (see Fig. S4 in the supplemental material). We infer that BRCA1/BARD1 probably functions in a pathway involving ATM and ATR kinases to promote ICL-induced G_2_ arrest.

A key effector in the G_2_/M checkpoint is the CHK1 kinase. CHK1 functions downstream of ATR in the checkpoint pathway and is activated upon DNA damage by its phosphorylation on serine 345. It has been proposed that BRCA1 is required to activate CHK1 in response to IR-induced DNA damage (12).

**FIG 5:**
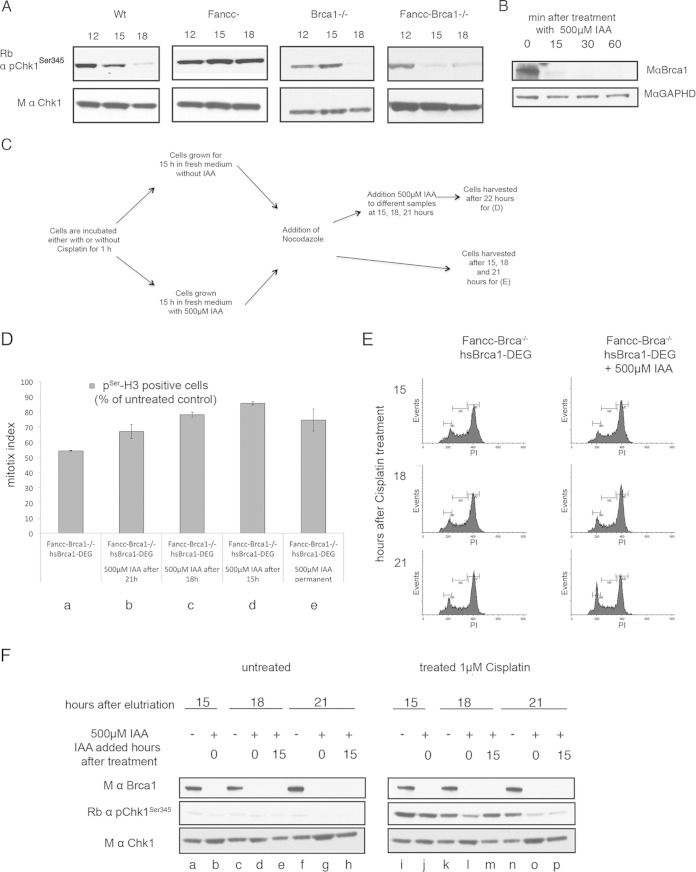
BRCA1 is required to maintain CHK1 activation in response to DNA damage. (A) CHK1 is activated through phosphorylation at serine 345 in response to cisplatin-induced DNA damage in wild-type and mutant cell lines (as indicated). A G_1_ population of cells for each mutant cell line was collected by centrifugal elutriation and treated with cisplatin for 1 h. The cells were incubated for the indicated times and analyzed for the presence of unmodified and phosphorylated (activated) CHK1 by Western blotting. (B) BRCA1-tagged with an auxin-inducible degron sequence (BRCA1-DEG) was expressed as a transgene in *Fancc*^−^ *Brca1*^−/−^ cells. Depletion of BRCA1-DEG with time after addition of 500 μM IAA was measured by Western blotting. Approximately 90% of the BRCA1-DEG protein was depleted within 15 min. (C) Schematic diagram depicting the experiments described in panels D and F. A 1 μM concentration of cisplatin was added to *Fancc*^−^ *Brca1*^−/−^ cells for 1 h at time zero, and the cells were grown for a further 22 h before harvesting them for analysis as described in panels D and F. BRCA1 was depleted in different samples of cells at 0, 15, 18, and 21 h by the addition of 500 μM IAA. (D) Depletion of BRCA1 correlates with impaired cell cycle arrest and progression into mitosis. *Fancc*^−^ *Brca1*^−/−^ cells expressing a Brca1-DEG transgene arrest in G_2_, as indicated by a decrease in the mitotic index. Depletion of BRCA1-DEG at 0, 15, 18, or 21 h after DNA damage results correlates with progression into mitosis as indicated by increased mitotic index. G_1_-synchronized *Fancc*^−^ *Brca1*^−/−^ cells plus BRCA1-DEG cells were treated with cisplatin for 1 h and then incubated for 22 h in fresh media. Next, 500 μM IAA was added to different samples. Cells were harvested after 22 h, and the mitotic index was determined (as described above). The data presented are the means from three experiments; error bars indicate one standard deviation. (E) Cell cycle profile of cisplatin-treated cells in panel D after the addition of IAA. Cells were fixed and stained with propidium iodide and analyzed by FACS to determine the DNA content. (F) Phosphorylation of CHK1 at Ser345 was determined in cell populations treated as for panel D. Loss of pCHK1 correlates with progression of cells into mitosis as determined by mitotic index (described above).

We next investigated whether BRCA1 was also required for the activation of CHK1 in response to DNA cross-link damage, or whether the kinetics of CHK1 activation was altered in these cells. To do this, we isolated synchronous populations of different mutant cells in G_1_ phase using centrifugal elutriation, treated them with 1 μM cisplatin for 1 h and measured the activation of CHK1 over time by Western blotting using antibody against pCHK1(ser345) (Fig. 5A). As expected, wild-type cells had elevated levels of pCHK1(ser345) 12 h after cisplatin treatment, which diminished by 15 h. In *Fancc*^−^ cells, CHK1 was also activated but persisted at 18 h, reflecting their failure to repair DNA damage. Interestingly, pCHK1(ser345) was also observed 12 h after cisplatin treatment in *Brca1*^−/−^ and *Fancc*^−^ *Brca1*^−/−^ cells, but, unlike *Fancc*^−^ mutant cells, this was largely diminished after 18 h. This suggested that initial activation of CHK1 through phosphorylation of serine 345 was not defective in *Brca1*^−/−^ and *Fancc*^−^ *Brca1*^−/−^ cells, and it did not explain the failure of these mutant cells to arrest in G_2_ phase. Instead, the progression of *Brca1*^−/−^ and *Fancc*^−^ *Brca1*^−/−^ cells into mitosis seemed to correlate with a failure to maintain pCHK1(ser345).

To investigate the contribution of BRCA1 to the maintenance of pCHK1(ser345), we used the auxin-inducible degron system, generating BRCA1 protein that could be rapidly depleted upon addition of the plant auxin indole-3-acetic acid (IAA) (38). We fused BRCA1 to an auxin-inducible degron (BRCA1-DEG) and introduced it as a transgene into *Fancc*^−^ *Brca1*^−/−^ mutant DT40 cells. We next confirmed that >90% BRCA1-DEG protein was depleted in cells 15 min after addition of 500 μM IAA (Fig. 5B). To measure the consequences of rapid BRCA1 depletion, we treated a synchronized G_1_ culture of *Fancc*^−^ *Brca1*^−/−^ cells expressing the BRCA1-DEG transgene with cisplatin for 1 h in the presence or absence of IAA and monitored their progression through the cell cycle as described previously (Fig. 5C). We confirmed that, in the absence of IAA, *Fancc*^−^ *Brca1*^−/−^ expressing BRCA1-DEG behaved as *Fancc*^−^ mutant cells, arresting in G_2_ phase 15 h after cisplatin treatment (Fig. 5D and E). These cells also had elevated levels of pCHK1(ser345) at 15 h, which persisted 22 h posttreatment (Fig. 5F, lanes i, k, and n), confirming that the BRCA1-DEG transgene was functional.

We then repeated the experiment, including 500 μM IAA in the medium throughout, to deplete BRCA1-DEG. As expected, depletion of BRCA1-DEG resulted in the failure of cells to accumulate in G_2_ with no observed decrease in mitotic index, mirroring the behavior of *Fancc*^−^ *Brca1*^−/−^ mutant cells (Fig. 5D, column e). Moreover, while the level of pCHK1(ser345) in these cells was elevated 15 h after cisplatin treatment, it was significantly diminished after 18 to 21 h (Fig. 5F, lanes j, l, and o), confirming impaired cell cycle arrest in these cells. It was again of note that constitutive depletion of BRCA1 throughout the experiment did not impair the initial activation of CHK1 by phosphorylation of serine 345, but the persistence of pCHK1(ser345) was affected at later time points.

To determine whether loss of BRCA1 function is directly linked to exit from cell cycle arrest in G_2_, we treated a G_1_ population of *Fancc*^−^ *Brca1*^−/−^ cells expressing BRCA1-DEG with cisplatin for 1 h and incubated them for a further 22 h to permit the cells sufficient time to arrest in G_2_ (Fig. 5E). During this incubation, we added 500 μM IAA to different samples at different time points—15, 18, and 21 h—in order to deplete BRCA1. The cells were harvested at 22 h, and the mitotic index was determined. We found that depletion of BRCA1 resulted in a clear time-dependent increase in mitotic index, with most cells progressing into mitosis within 6 h after BRCA1 depletion (Fig. 5D, compare columns a to d). Moreover, G_2_ exit coincided with a rapid and significant reduction in pCHK1(ser345) (Fig. 5F, compare lanes l, m, and p). This contrasted sharply with the cell cycle arrest and persistence of pCHK1(ser345) in cells expressing functional BRCA1.

We conclude that BRCA1, BARD1, and ABRAXAS help maintain cell cycle arrest/delay in FA cells treated with cisplatin and that this requirement persists many hours after the initial exposure to DNA damage. This failure to arrest correlates with improved survival of FA cells to low levels of DNA cross-link damage.

## DISCUSSION

BRCA1 has been implicated in the repair of interstrand DNA cross-links by promoting homology-dependent repair of DNA breaks in the FA pathway (4, 10). We present evidence that, in DT40, BRCA1/BARD1 may play another important role, by helping to maintain G_2_/M phase cell cycle arrest in FA cells with unrepaired DNA damage.

We report that cell cycle arrest and subsequent apoptotic cell death, which occurs in FA-defective DT40 cells after treatment with DNA cross-linking agents, is lost in BRCA1-deficient cells. Instead, cisplatin-treated FA cells lacking functional BRCA1 transit through G_2_ phase and mitosis with unrepaired DNA damage, leading to an increase in overall viability.

Our initial observation that *Brca1*^−/−^ DT40 cells are not very sensitive to cisplatin-induced DNA damage is at variance with studies in mammalian cancer cell lines. Nevertheless, we confirmed our observations using independently derived *Brca1*^−/−^ mutant cell lines (data not shown). It is possible that avian and mammalian cells deal differently with DNA cross-link damage. However, this is unlikely, because DT40 has previously provided an excellent model for the FA pathway in vertebrates. Alternatively, there might be differences in the DNA damage response (DDR) between lymphocytes and epithelial cancer cells. Finally, the discrepancy might reflect differences in genetic background of these cell lines, which is known to have a profound influence on sensitivity to DNA damage (as discussed above). Nevertheless, despite the overlapping biological mechanisms of the FA and BRCA1 pathways in DT40 and human cells, the clinical relevance of these findings in cancer should be treated with caution.

That *Fancc*^−^ *Brca1*^−/−^ DT40 cells are defective for the repair of DNA cross-link damage but are minimally sensitive to 1 μM cisplatin treatment, is reconciled by our demonstration that defects in BRCA1 impair cross-link-induced G_2_/M checkpoint activity. We note that this is most apparent after relatively short exposure (1 h) to moderate levels of cisplatin, where cell death occurs largely through apoptosis as part of the DDR, rather than through catastrophic mitosis. The link between repair of DNA lesions and cell cycle progression is critical to prevent the proliferation of cells with unrepaired DNA damage. Our data indicate that BRCA1 plays a key role to promote DDR-mediated cell death in cells with unrepaired cross-link damage. It is likely that the combination of defective DSB repair and failure to promote death in BRCA1^−/−^ cells with unrepaired DNA damage accounts for the profound increase in chromosomal aberrations observed in these cells (10).

The resolution of DNA cross-link damage is multifaceted, involving interplay between several DNA repair pathways. Several years ago, the Patel and La Volpe groups reported that suppression of NHEJ in FA cells resulted in increased resistance to treatment with DNA cross-linking agents. These researchers hypothesized that the suppression of NHEJ promoted the accurate repair of DNA breaks through HR, rather than abortive and toxic repair that ensues when NHEJ is functional (12, 24, 25). Our data indicate that the relationship between the FA pathway and BRCA1 is different, with resistance to DNA cross-link damage promoted through abrogation of DNA damage-induced checkpoint function, rather than interplay between potentially conflicting repair pathways.

BRCA1 has previously been implicated in the G_2_/M checkpoint in response to IR (11). A key effector in IR-induced cell cycle arrest is the checkpoint kinase Chk1, which is activated through phosphorylation on serine residue 345. Yarden et al. reported that BRCA1-defective HCC1937 cells fail to arrest in G_2_/M after treatment with IR and that reexpression of BRCA1 in these cells restored IR-induced G_2_ arrest with a concomitant increase in the activation Chk1 (12). It was subsequently shown that BRCA1 contributes directly to the phosphorylation of Chk1 by promoting its association with claspin (12, 39). Together, these studies indicated a role for BRCA1 in the initiation of onset of IR-induced checkpoint signaling by promoting activation of Chk1.

This is clearly not the case in DT40 treated with DNA cross-linking agents. *Fancc*^−^ *Brca1*^−/−^ and *Brca1*^−/−^ mutant cells initiate normal checkpoint signaling after cisplatin treatment with concomitant increased levels of pCHK1(ser345). Instead, we found that BRCA1 is required to prolong G_2_ arrest and that depletion of BRCA1 coincides with rapid exit of cells from G_2_ and entry into mitosis, with a significant reduction in pCHK1(ser345). Therefore, BRCA1 is required to maintain checkpoint function many hours after the initial exposure to DNA damage. It is currently not clear whether BRCA1 contributes directly to the maintenance of the pCHK1(ser345) signal or if the pCHK1(ser345) diminishes as a consequence of entry into mitosis as the checkpoint is relieved. Recently, Venkitaraman and coworkers identified a role for ubiquitin ligase function of BRCA1 in the activation of Chk1 after treatment with topoisomerase poisons but not with mitomycin C (40). Although our data indicate that BRCA1 might be required to maintain Chk1 activation in response to DNA cross-link damage, we have observed no dependence on the E3 function of BRCA1 (data not shown).

In cells treated with the replication inhibitor aphidicolin, Chk1 is required to maintain viable replication structures, and it is the persistence of these structures, rather than Chk1 activation *per se*, which maintains the checkpoint until replication is complete (41). Our data are consistent with a similar model in the repair of DNA cross-link damage, where stalled forks induced by DNA cross-linking damage are stabilized and maintained by Chk1, pending their repair by the FA pathway. Failure to repair these structures eventually leads to their degeneration and subsequent loss of checkpoint signal (41). In this model, defects in BRCA1 might provide a link between preservation of stalled replication forks and their ability to restart replication after DNA damage. Lack of BRCA1 might lead to the premature degeneration of stalled replication structures, the loss of Chk1 signaling, and aberrant entry into mitosis.

This is supported by work from Ceccaldi et al. (42), who described a group of FA patients with attenuated phenotype. Cells from attenuated FA patients exhibit characteristics very similar to those reported here for *Fancc*^−^ *Brca1*^−/−^ DT40 mutants (42). Although these patients had nearly normal blood counts, their peripheral blood lymphocytes failed to arrest in G_2_ phase after treatment with mitomycin C. Their cells also exhibited numerous chromosomal breaks. Although the levels of pChk1(ser345) were not quantified in these patients, they did exhibit lower than normal levels of Chk1. Importantly, the defective G_2_ checkpoint linked to cellular proliferation with increased genome stability observed in patients' cells directly mirrors our findings with FA-defective DT40 cells lacking BRCA1. Ceccaldi et al. suggested that the enhanced genome instability observed in attenuated FA patients might contribute to the subsequent development of leukemia in these patients.

Recently, Bunting et al. showed that *Brca1*^Δex11/Δex11^ *53bp1*^−/−^ cells are proficient in homologous recombination but sensitive to ICL damage. This indicated that, in addition to its function in HR, BRCA1 has a role in cross-link repair (9). The authors suggest that BRCA1 helps to promote the localization of FANCD2 to chromatin at sites of DNA damage. However, it is not clear why failure to efficiently recruit FANCD2 to sites of DNA damage would not also impact on downstream homologous recombination events. Our data provide another explanation by showing that BRCA1 is required to maintain G_2_ arrest in FA cells. This also explains the reduced levels of FANCD2 nuclear foci observed in BRCA1-defective cells exposed to ICL. Since the relocalization of FANCD2 occurs mainly in the S and G_2_ phases of the cell cycle, the escape of BRCA1-defective cells from G_2_ phase and their progression into mitosis would reduce in the proportion of cells exhibiting FANCD2 foci.

BRCA1 interacts with several partner proteins in a cell cycle dependent manner through its BRCT domains. Of these, we have identified a role for ABRAXAS in checkpoint signaling in FA-deficient cells. Like *Fancc*^−^ *Brca1*^−/−^ mutants, *Fancc*^−^ *Abra1*^−/−^ cells exhibit increased cisplatin resistance and failure to maintain pCHK1(ser345) levels in G_2_ compared to FANCC-defective cells. On the other hand, defects in BRIP1 are not linked with these phenotypes, indicating that components of the BRCA1-A complex but not of the BRCA1-B complex contribute this G_2_ checkpoint function. Future studies will be required to determine whether BRCA1-A plays a direct or indirect role in the maintenance of Chk1 activation. However, it is noteworthy that both DT40 and human cells that are defective in ABRAXAS are proficient in homologous recombination. Since *Abra1*^−/−^ cells do not maintain G_2_ arrest, we infer that its function in ICL-induced G_2_ arrest is genetically separable form homologous recombination. Hence, our observations might provide an explanation for the homologous recombination-independent function of BRCA1 reported by Bunting et al. and explain how the loss of BRCA1 can promote the proliferation and survival of FA cells harboring DNA damage.

Together, our data uncover an important role for BRCA1 in the FA pathway in DT40 by promoting the maintenance of DNA-cross-link-induced G_2_ arrest many hours after exposure to DNA damage. This function provides quality control for FA cells entering into mitosis and promotes apoptotic death in cells with persistent unrepaired DNA damage. Moreover, it is clear that impaired DNA repair function coupled with loss of G_2_ checkpoint function might have clinical implications for the treatment of attenuated FA patients.

## Supplementary Material

Supplemental material
