# Supplementary material for: BRCA1 Is Required for Maintenance of Phospho-Chk1 and G2/M Arrest during DNA Cross-Link Repair in DT40 Cells
Source: Mol Cell Biol. 2015 Oct 16;35(22):3829–40. doi: 10.1128/MCB.01497-14 (PMC4609749; doi:10.1128/MCB.01497-14)
Supplement: Supplemental material [file supp_35_22_3829__index.html]

BRCA1 Is Required for Maintenance of Phospho-Chk1 and G2/M Arrest during DNA Cross-Link Repair in DT40 Cells — Supplemental material 

# BRCA1 Is Required for Maintenance of Phospho-Chk1 and G2/M Arrest during DNA Cross-Link Repair in DT40 Cells

## Supplemental material

- Supplemental file 1 -

  Fig. S1 (*Fancc*− cells after treatment with cisplatin), S2 (Cell cycle profiles of untreated DT40 cell lines), S3 (BRCA1 and FANCC in repair of interstrand cross-links), and S4 (Fancc-Brca1−/− HsBRCA1 cells after treatment with cisplatin)

  PDF, 4.9M
