# Supplementary material for: BRCA1 Is Required for Maintenance of Phospho-Chk1 and G2/M Arrest during DNA Cross-Link Repair in DT40 Cells
Source: Mol Cell Biol. 2015 Oct 16;35(22):3829–40. doi: 10.1128/MCB.01497-14 (PMC4609749; doi:10.1128/MCB.01497-14)
Supplement: Supplemental material [file MCB.01497-14_zmb999101006so1.pdf]

BRCA1 is required for maintenance of phospho-Chk1 and G2/M arrest during DNA crosslink repair in DT40 cells.

Margarethe Draga, Elizabeth B. Madgett, Cassandra J. Vandenberg, David du Plessis, Aisling Kaufmann, Petra Werler, Prasun Chakraborty, Noel F. Lowndes and Kevin Hiom*.

* To whom correspondence should be addressed.

E mail: k.hiom@dundee.ac.uk

This file contains: Supplemental figures S1-S4

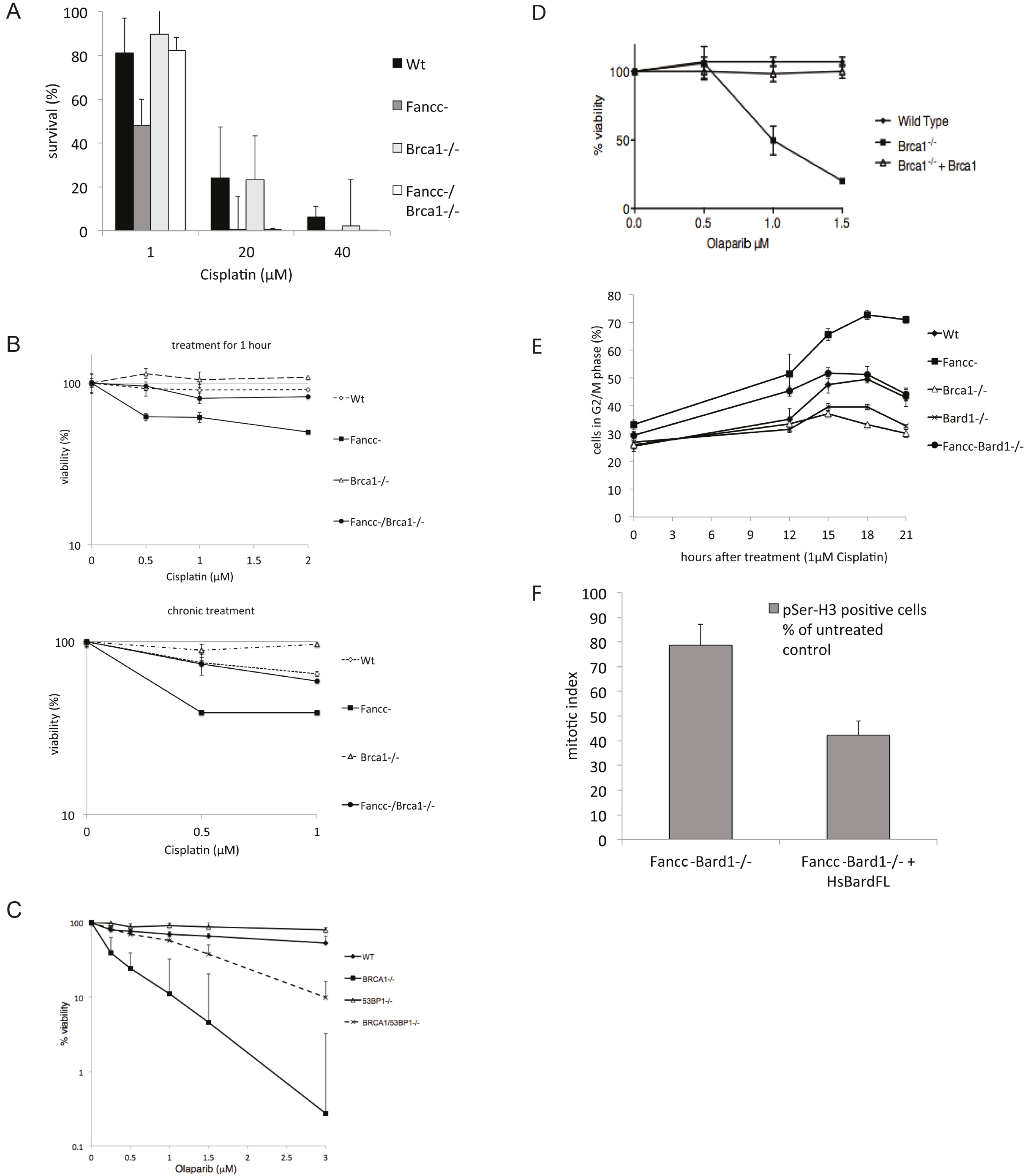

A
survival (%)
100
80
60
40
20
0
1
20
40
Cisplatin (μM)
Wt
Fancc-
Brca1-/-
Fancc-/
Brca1-/-
B
treatment for 1 hour
viability (%)
100
10
0
0.5
1
1.5
2
Cisplatin (μM)
Wt
Fancc-
Brca1-/-
Fancc-/Brca1-/-
chronic treatment
viability (%)
100
10
0
0.5
1
Cisplatin (μM)
Wt
Fancc-
Brca1-/-
Fancc-/Brca1-/-
C
% viability
100
10
1
0.1
0
0.5
1
1.5
2
2.5
3
Olaparib (μM)
WT
BRCA1-/-
53BP1-/-
BRCA1/53BP1-/-
D
% viability
100
50
0
0.0
0.5
1.0
1.5
Olaparib μM
Wild Type
Brca1-/-
Brca1-/- + Brca1
E
cells in G2/M phase (%)
80
70
60
50
40
30
20
10
0
0
3
6
9
12
15
18
21
hours after treatment (1μM Cisplatin)
Wt
Fancc-
Brca1-/-
Bard1-/-
Fancc-Bard1-/-
F
mitotic index
100
90
80
70
60
50
40
30
20
10
0
pSer-H3 positive cells
% of untreated
control
Fancc-Bard1-/-
Fancc-Bard1-/- +
HsBardFL

Figure S1: *Fancc*$^{-}$ mutant cells, but not *Brca1*$^{-/-}$, *Bard1*$^{-/-}$, *Fancc-Brca1*$^{-/-}$ or Fancc^{-}*Bard1*$^{-/-}$ cells arrest in G2 after treatment with cisplatin.

(A) Methylcellulose Colony Survival assays were carried out after treatment of cells with different concentrations of Cisplatin. *Brca1*$^{-/-}$ and *Fancc*$^{-}$*Brca1*$^{-/-}$ are less sensitive than *Fancc*$^{-}$ mutant cells to treatment with 1μM Cisplatin. However, *Fancc*$^{-}$*Brca1*$^{-/-}$ and *Fancc*$^{-}$ exhibit similar sensitivities after treatment with higher concentration (20-40 μM) Cisplatin, (B) MTS cell proliferation assays were carried out 48 hours after either 1h or chronic treatment of cells with different concentrations of Cisplatin. *Brca1*$^{-/-}$ and *Fancc-Brca1*$^{-/-}$ are less sensitive to treatment than *Fancc*$^{-}$. The mean data of three experiments is shown. (C) Methylcellulose colony survival assays were carried out after treatment of cells with different concentrations of the Parp inhibitor Olaparib. Sensitivity of *Brca1*$^{-/-}$ DT40 mutant cells is significantly corrected in *53BP1*$^{-/-}$ *Brca1*$^{-/-}$ mutant cells. (D) MTT cell viability assays of indicated DT40 wild type, mutant and corrected mutant cell lines as indicated. Graphs show that sensitivity of *Brca1*$^{-/-}$ DT40 cells to treatment with the PARP inhibitor Olaparib is reversed by expression of Brca1 transgene. (E) Wild type and mutant cells were damaged with 1μM cisplatin, pulse-labeled with BrdU and harvested at times indicated. Cells were analyzed for BrdU incorporation and Propidium iodide staining by FACS to determine DNA content. Percentage of cells with near 4C DNA content and therefore in G2/M phase of the cell cycle are shown at different times after DNA damage treatment. (E) *Fancc*$^{-}$*Bard1*$^{-/-}$ mutant cells that are reconstituted with full-length Hs Bard1 (FL), but not *Fancc*$^{-}$*Bard1*$^{-/-}$ cells arrest prior to entering mitosis after treatment with cisplatin. Mitotic index of cells treated with 1μM cisplatin is shown. Nocodazole was added 15 hours

after damage treatment to trap cells entering mitosis. Mitotic cells were quantified 24 hours after treatment by staining for pSer-H3 and measured by FACS. Mitotic index is calculated as the ratio of treated/untreated cells staining positive for pSer-H3.

A

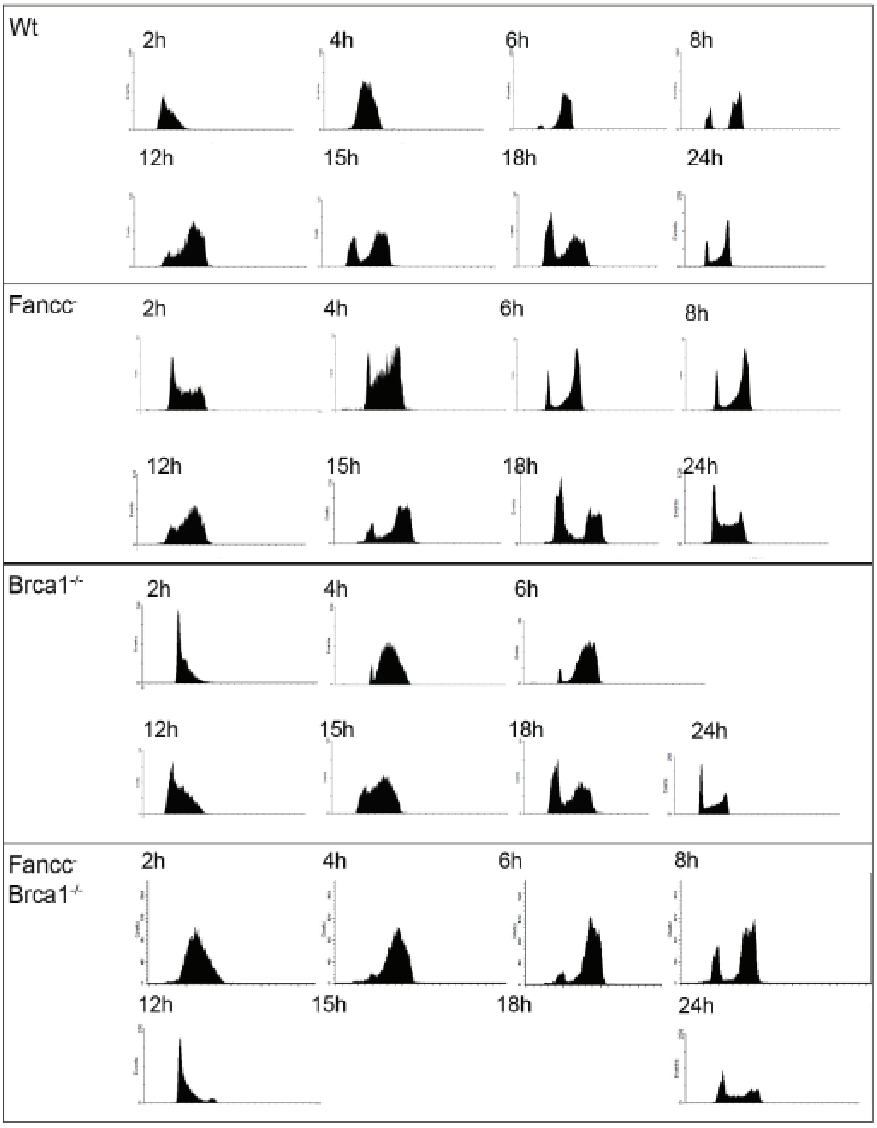
Wt
2h
4h
6h
8h
12h
15h
18h
24h
Fancc-
2h
4h
6h
8h
12h
15h
18h
24h
Brca1-/-
2h
4h
6h
12h
15h
18h
24h
Fancc-
Brca1-/-
2h
4h
6h
8h
12h
15h
18h
24h

B

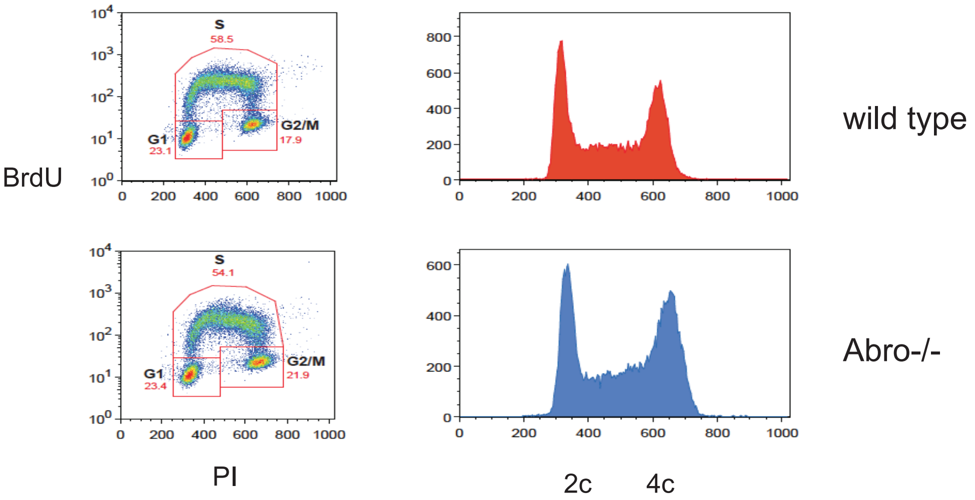
BrdU
S
58.5
G1
23.1
G2/M
17.9
wild type
S
54.1
G1
23.4
G2/M
21.9
Abro-/-
PI
2c
4c

Figure S2: Cell cycle profiles of untreated DT40 cell lines.

(A) Populations of G1 cells were collected using centrifugal elutriation and incubated for different times. Samples were fixed and stained with propidium iodide after the times indicated and DNA content analysed by FACS. (B) Asynchronous untreated populations of wild type and Abra-/- cells were pulse-labeled with BrdU, harvested and stained with propidium iodide (PI). Cells were analyzed for BrdU incorporation and PI staining using FACS.

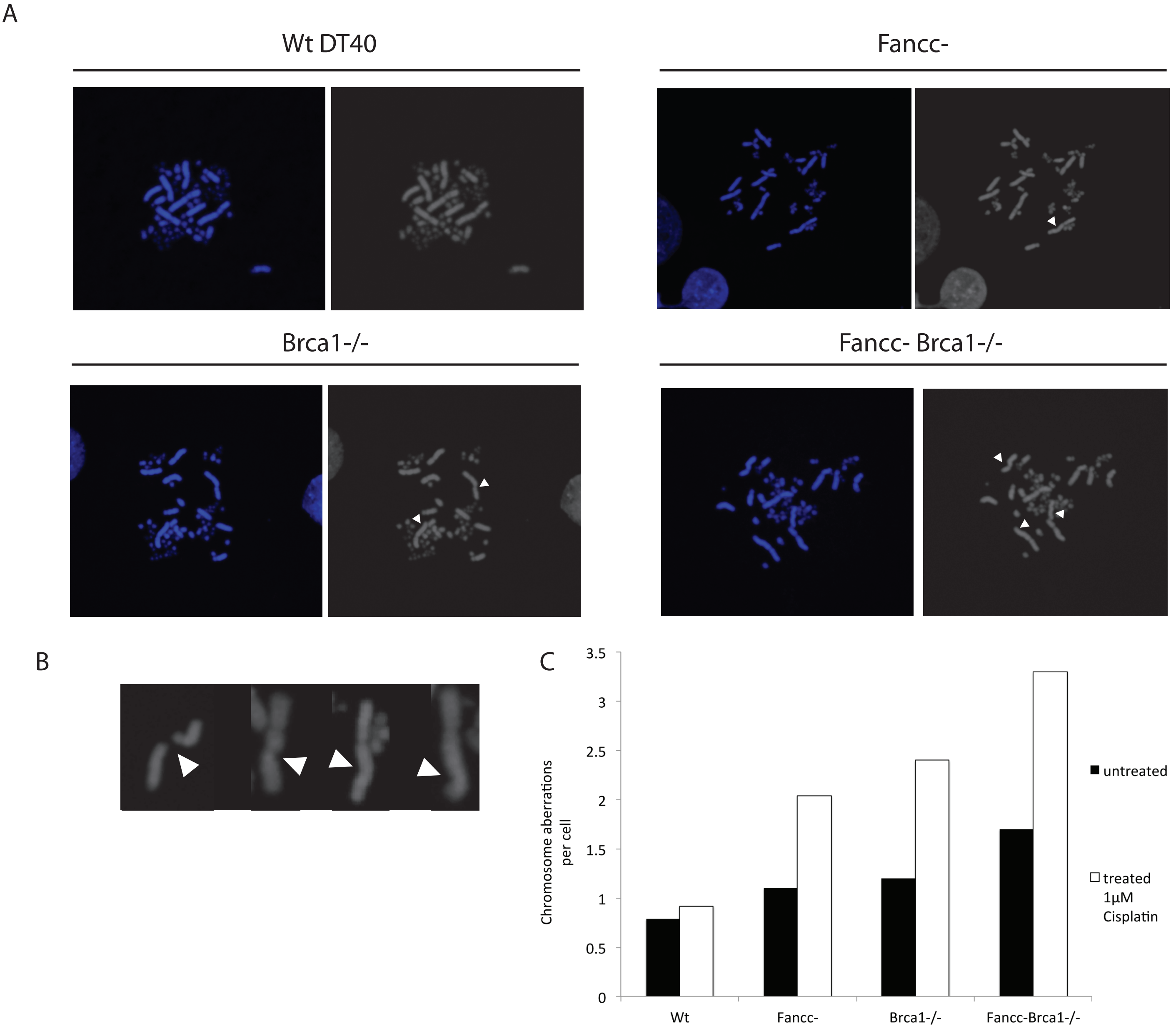
A
Wt DT40
Fancc-
Brca1-/-
Fancc- Brca1-/-
B
C
Chromosome aberrations per cell
3.5
3
2.5
2
1.5
1
0.5
0
Wt
Fancc-
Brca1-/-
Fancc-Brca1-/-
untreated
treated 1μM Cisplatin

Figure S3: BRCA1 and FANCC are required for the repair of interstrand crosslinks.

(A) Representative metaphase spreads stained with DAPI of wild type, *Brca1*$^{-/-}$, *Fancc*$^{-}$ and *Fanc-Brca1*$^{-/-}$ mutant DT40 cells. Arrowheads indicate breaks in the macro-chromosomes. (B) Enlargement of examples for counted chromosome breaks. (C) Chromosome break analysis of wild type, *Brca1*$^{-/-}$, *Fancc*$^{-}$ and *Fanc-Brca1*$^{-/-}$ mutant DT40 cells. Cells were incubated for 1 hour with 1µM Cisplatin, left to recover for 15 hours, treated with 0.1µg/ml Colcemid for 1.30-2hours. Preparation of Metaphase spreads is described in Methods and Materials. The chromosome breaks of 100 metaphases were counted; Data shown are the mean of two experiments.

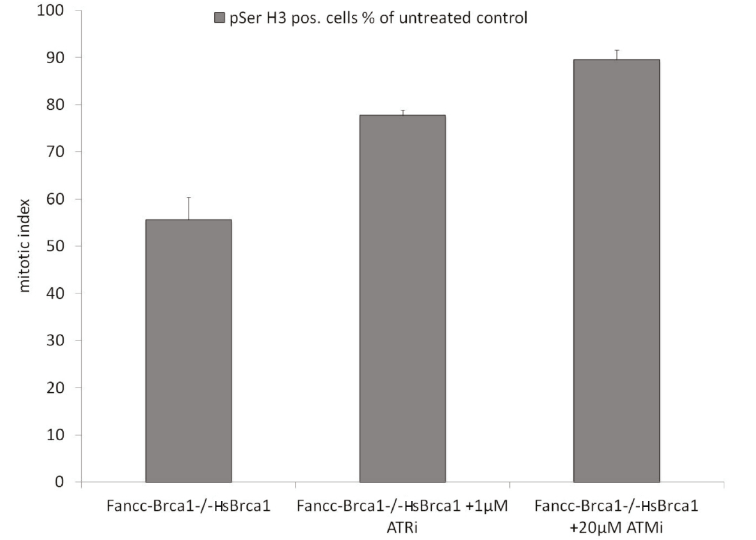
pSer H3 pos. cells % of untreated control
mitotic index
0
10
20
30
40
50
60
70
80
90
100
Fancc-Brca1-/-HsBrca1
Fancc-Brca1-/-HsBrca1 +1μM ATRi
Fancc-Brca1-/-HsBrca1 +20μM ATMi

Figure S4: Fancc-Brca1-/- + HsBRCA1 cells arrest prior to entering mitosis after treatment with cisplatin.

Inhibition of ATM and ATR as well as the treatment with IAA improve cell cycle progression into mitosis. Mitotic index of cells treated with 1µM cisplatin is shown. The inhibitors ATM (KU55933) and ATR (ETP 46464) or IAA were added 15 hours after damage treatment. Furthermore nocodazole was added to trap cells entering mitosis. Mitotic cells were quantified 24 hours after treatment by staining for pSer-H3 and measured by FACS. Mitotic index is calculated as the ratio of treated/untreated cells staining positive for pSer-H3.

Data presented are the mean of two experiments; error bars indicate one standard deviation
